# Fogging Control on LDPE/EVA Coextruded Films: Wettability Behavior and Its Correlation with Electric Performance

**DOI:** 10.3390/membranes7010011

**Published:** 2017-02-22

**Authors:** Miguel A. Waldo-Mendoza, Zoe V. Quiñones-Jurado, Juan C. Pérez-Medina, Bernardo Yañez-Soto, Pedro E. Ramírez-González

**Affiliations:** 1 Innovación y Desarrollo en Materiales Avanzados A. C., Grupo POLYnnova, Carr. San Luis Potosí-Guadalajara 1510, Nivel 3, Local 12, Lomas del Tecnológico, San Luis Potosí 78211, Mexico; jcarlospm40@hotmail.com (J.C.P.-M.); pedro.e.ramirez.g@gmail.com (P.E.R.-G.); 2 Instituto de Física “Manuel Sandoval Vallarta”, Universidad Autónoma de San Luis Potosí, Álvaro Obregón 64, San Luis Potosí 78000, Mexico; byanez@ifisica.uaslp.mx

**Keywords:** AC electrical conductivity, wettability, nonionic surfactants, ethylenevinyl acetate, low-density polyethylene, coextruded films

## Abstract

The transformation of fog at a non-visible water layer on a membrane of low-density polyethylene (LDPE) and ethylene-vinyl acetate (EVA) was evaluated. Nonionic surfactants of major demand in the polyolefin industry were studied. A kinetic study using a hot fog chamber showed that condensation is controlled by both the diffusion and permanency of the surfactant more than by the change of the surface energy developed by the wetting agents. The greatest permanency of the anti-fog effect of the LDPE/EVA surface was close to 3000 h. The contact angle results demonstrated the ability of the wetting agent to spread out to the surface. Complementarily, the migration of nonionic surfactants from the inside of the polymeric matrix to the surface was analyzed by Fourier transform infrared (FTIR) microscopy. Additionally, electrical measurement on the anti-fogging membrane at alternating currents and at a sweep frequency was proposed to test the conductivity and wetting ability of nonionic surfactants. We proved that the amphiphilic molecules had the ability to increase the conductivity in the polyolefin membrane. A correlation between the bulk electrical conductivity and the permanency of the fogging control on the LDPE/EVA coextruded film was found.

## 1. Introduction

Wettable surfaces have become of great interest in applications such as biocompatibility, printing, coatings, food technology, optical devices, hygienic industries, etc. [1]. For agricultural applications, the development of wettable surfaces has been extensively demanded in the design of greenhouse membranes. Particularly for these films, the durability of the wetting effect is desired to be long-lasting [1]. The usual solution, due to economic and practical reasons, is the employment of low-density polyethylene-based films with improved wettability. It is well known that the hydrophobic effect is conventionally attributed to the polyolefin plastic. For overcoming the poor affinity with water, the surface energy of the polyolefin must be increased to improve the wetting and to avoid the formation of water drops on the surface in contact with moisture [2]. Nonionic surfactants are usually employed for the modification of polyolefin surfaces in order to increase the moisture affinity [1,2,3]. A significant aspect of a surfactant is its great ability to lower the interfacial tension between a solid phase with a liquid or vapor water, due to its amphiphilic character [4]. However, the correct combination of efficient wettability and reliability is one of the most important challenges for the technological development of polyolefins [5].

Since the effect of the surfactant is not permanent, it is easily extracted from the surface that it keeps moist. There have been some attempts to substitute the use of surfactants. Several hydrophilic compounds have been developed as non-migratory agents, including silicon-based molecules, metallic oxide nano-particles and blends with hydrophilic polymers, among others [1,6,7,8,9,10,11,12]. Current technology that increases the wettability of non-polar polymers using surfactants is preferred by the industry, because it implies minor technological difficulties and lower costs without alterations in the bulk properties of the polyolefin [13,14]. However, its performance is limited to the migration of the additive from the inside of the polymeric matrix to the surface in contact with water and also to the retention of released amphiphilic molecules [15].

Despite nonionic surfactants being extensively used, a systematic comparison between their performance is pertinent in order to determine the best compound in terms of efficiency and durability. The aim of this work is to show how the diffusion and wetting capacity of an amphiphilic agent can be controlled by a membrane with EVA. In addition to the superficial characterization by the mean contact angle (MCA) and the condensation kinetics by the hot fog test, we propose the use of electrical measurements to show the correlation of the wetting behavior which can be achieved as time passes with the electrical conductivity of the studied materials.

## 2. Results

### 2.1. Wetting of the LDPE/EVA Coextruded Film by Effect of the Different Nonionic Surfactants

In order to test the diffusion rate of each additive and its wetting ability, we produced all the films with a polarity asymmetry between layers A and B, where layer A was formed with EVA (with a final amount of 15% of vinyl acetate groups) and layer B with LDPE polymer. Two kinds of membranes were produced by coextrusion, type I with 3 wt % of nonionic surfactant in layer A and type II with 5 wt %. Both types (I and II) contained only 1 wt % of surfactant in layer B. The studied nonionic surfactants were: glycerol ester (GE), octadecanoic acid (OA), polyglycerol ester (PE), polyglycerol stearate;1,2,3-propanetriol (PS), polyglycerol monostearate (PM), sorbitan monostearate (SM), glyceril monooleate (GM).

Mean contact angle (MCA) measurements were performed for each coextruded film on both layers and were compared with the film without nonionic surfactants as a blank. Table 1 presents the MCA value of the coextruded films on sides A and B, containing 3% and 1% of the surfactant concentration, respectively. The MCA results were processed using a two-way analysis of variance, for which variability was determined to be significant (α = 0.05) using a Bonferroni multi-comparison test. The statistical analysis shows a significant difference in the wettability between the blank membranes and the membranes with nonionic surfactants, implying a decrease of the MCA by the use of the additives. In contrast, no significant difference was between layer A and B of the blank, showing that the hydrophilic nature of the EVA copolymer with a final amount of 15 wt % of vinyl acetate groups does not have an impact on the affinity of the film with water.

The content of different amphiphilic additives enhanced the wetting. Film evaluation on side A showed MCA measurements from 6 to 48 degrees, and side B showed MCA measurements from 0 to 89 degrees for the different chemistries.

Regarding the membranes with nonionic surfactants, we report a non-significant difference between layers A and B for the compounds SM, GM, GE, and OA. The easiest form of rationalizing our observations is assuming that these particular agents migrate faster and the concentration asymmetry is balanced in a short time by the diffusion of the additive from the more- to the less-concentrated side. On the other hand, we found a significant difference between layers A and B of films produced with the following additives: PE, PS, and PM. An overall observation is that layer A was always more wettable than layer B. That difference is clearly related to the asymmetry amount imposed in the design of the films. Hence, the diffusion rate of these agents (PE, PS, and PM) was slow and the concentration gradient was balanced over a long time. In summary, the wetting results on film sides A/B are listed in decreasing order, as is indicated: OA > GE; GM; SM > PM; PE > PS (Table 1).

### 2.2. Diffusion of Amphiphilic Additives on Interface of the Membrane Formed with LDPE/EVA Layers

The amount of additive between layers imposes a gradient concentration which we initially considered could promote the diffusion of the wetting agent from layer A to layer B, due to the greater amount of additive in layer A (3 wt %) than in layer B (1 wt %). However, the force driving the transport was influenced by the hydrophilic affinity of the EVA layer. In the case of GM, PM, PE and PS additives, they were mainly expelled to the side A surface (Table 1). Besides, these measurements can be related with the capability of the EVA layer to retain the additives inside the polymeric matrix. The MCA result on the film surface was influenced by the chemistry of the used agent. However, it was also observed that the gradient concentration between layers A and layer B, apparently, strongly depended on the migration level of the wetting agent and it could be classified into two categories. As an example, we observed a difference of approximately 30° in the MCA between layer A and B for all the following additives: PE, PM and PS; because of that, we considered these additives as agents of slow release. For OA, GE, GM and SM additives, a MCA value was established between both layers smaller than 10°. Thus, we can assume that migration is faster and depends on a low molecular weight (Table 2) and low affinity with respect to both the EVA and LDPE polymers.

### 2.3. Effect of the Concentration of Nonionic Surfactants on the Wetting of LDPE/EVA Surfaces

**Figure 1:**
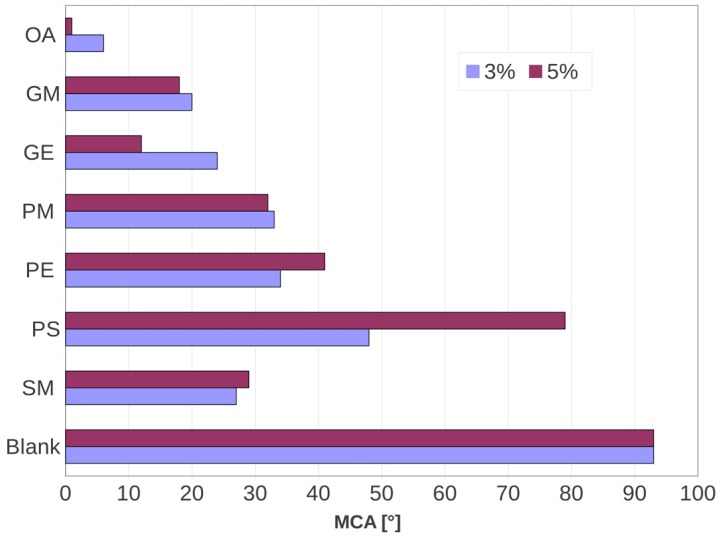
Comparison between the MCA results of tested films correspond to 3 and 5 wt % of nonionic surfactant concentration in layer A.

Type II membranes with a total amount of 5 wt % of nonionic surfactant in layer A were produced and tested. The correlation of the MCA values of those films with the type I membrane (3 wt % of additive in layer A) is shown in Figure 1.

MCA measurements did not show a considerable decrease when the nonionic surfactant amount was 5 wt %. For the OA and GE additives we observed a slight decrease of the MCA when the amount of additive was increased. In the SM, PM, PE and GM additives, a non-real change of the MCA was observed. Unfavorably, the wetting behavior did not get better for most of the nonionic surfactants at a 5 wt % concentration. For PS we found a MCA of 79° when 5 wt % was added to layer A, in comparison with the 48° recorded for the same additive but at a 3 wt % concentration.

The intuitive idea of assuming a better wetting performance for a higher amount of amphiphilic additive was proved wrong. Our MCA test results suggest, except for OA and GE, that the additive at a great amount can tend to form clusters or aggregates inside the polymer matrix. It is necessary to remember that all of these compounds are amphiphilic and have partial affinity with the LDPE film. In a similar way, the polar fraction of the surfactant can start to congregate when the concentration increases. An important aspect to consider is if the congregated additive or cluster is within the membrane or if the clusters are formed outside of the surface. In the first case, the wettability reduction could be related to the difficulty the clusters have in migrating, which implies the non-exposition of hydrophilic functionality in the formed aggregates. This behavior suggests that a better dispersion of the additive will surely promote the optimum placing of polar groups over the surface. Thus, we suppose that a small amount of additive could permit the polar groups to spread out of the polymeric material and to place themselves over the surface (Figure 1).

The possible formation of aggregates inside of the EVA layer was interpreted by FTIR image analysis. FTIR maps were collected of the surface corresponding to the film containing the PS surfactant, because it showed a low increase in the surface energy and the worst effect on the MCA at 5 wt %.

**Figure 2:**
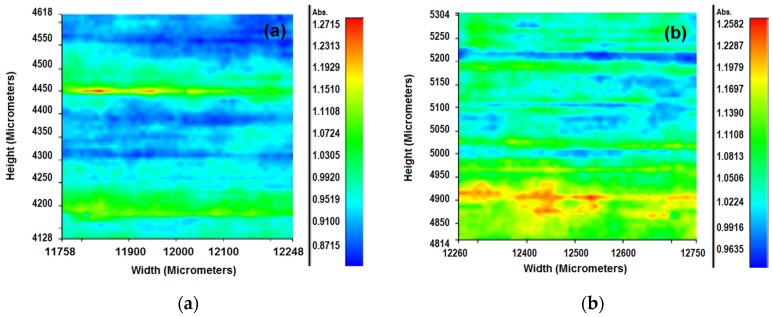
FTIR scanning of the superficial polar groups over the surface of films produced with PS surfactant at 5 wt % (**a**) and 3 wt % (**b**).

Figure 2 shows the comparison of the density of the polar groups on layer A of the films with 3 wt % and 5 wt % of PS surfactant. The color scale shown at the right of each panel indicates the concentration of the polar groups, the scale in red means a high concentration and blue means a low concentration. FTIR maps on layer A of the type II (5 wt %) membrane basically show a blue color surface and some green random zones (Figure 2a). Meanwhile, the FTIR maps of the type I membrane, with 3 wt % of PS in layer A show continuous zones mostly in green, which contain small areas in yellow and red colors (Figure 2b). The FTIR scanning of the films showed a better dispersion of the additive in the surface of films produced with only 3 wt % of the nonionic surfactant.

### 2.4. Evaluation of Condensation Kinetics of Water Vapor on LDPE/EVA Surfaces

**Figure 3:**
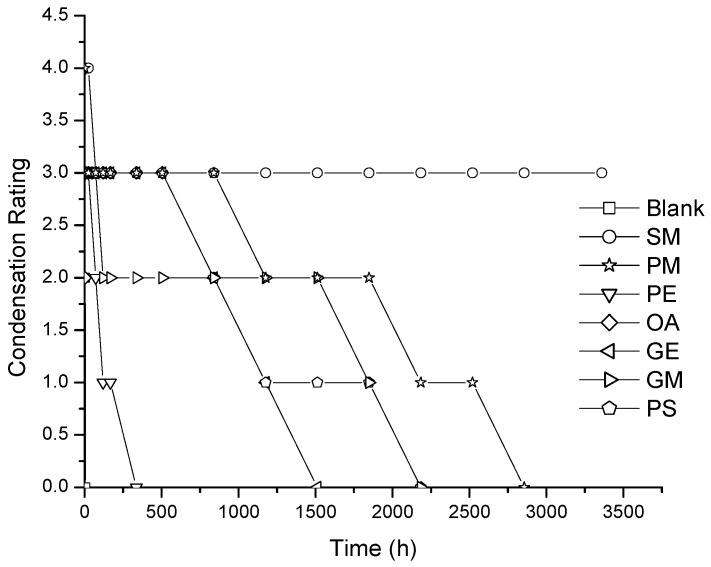
Condensation ratings for LDPE/EVA coextruded film containing 3 wt % of nonionic surfactant. For all the cases the layer (A) is facing the inner part of the chamber.

**Figure 4:**
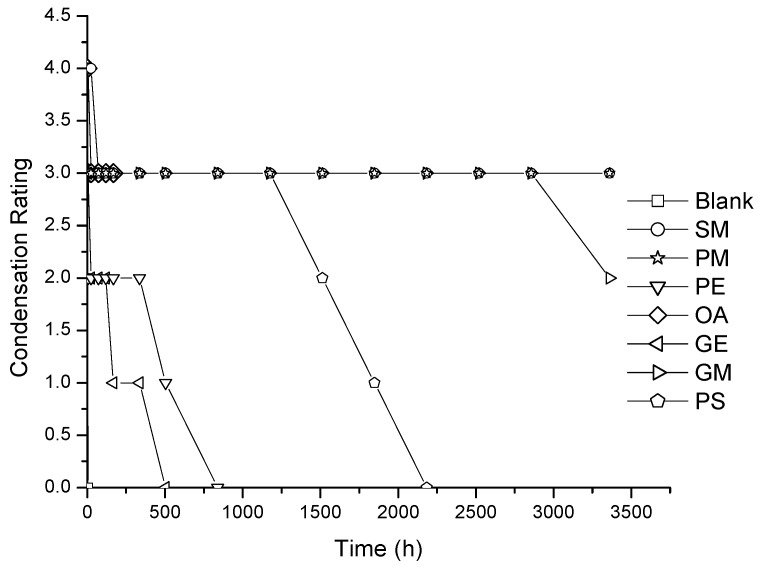
Condensation ratings for LDPE/EVA coextruded film containing 5 wt % of nonionic surfactant. For all the cases the layer (A) is facing the inner part of the chamber.

The evaluation of wetting over films under the hot fog chamber allowed us to correlate the nonionic surfactants’ diffusion and the effect of wetting over time. First, we present the results of the hot fog test using layer A of the membrane to the interior of the chamber (Figure 3 and Figure 4).

According to the literature, the rate that qualifies the best wetting additive for condensing water on plastic without affecting the transparency of the film is the one that maintains the 4 (excellent) rate for a longer time [1]. As expected, the blank film had a very poor performance (rating 0). Layer A of type I membranes (3 wt % of agent) showed an acceptable rating for SM and PM, close to 1000 and 1500 h, respectively. The GE additive only showed an acceptable rating until a time of 500 h, the rest decreased in a short time (<168 h) (Figure 3). On type II membranes with 5 wt % of nonionic surfactant, the best performance of layer A was achieved for about 3000 h for PM and GM, while the rest of the additives had an unacceptable effect, such as the blank film (layer containing EVA) (Figure 4). This result suggests that additive clustering exists inside the polymeric membrane which contributes to controlling the diffusion of the wetting agent to the film surface, increasing the wetting efficiency for a longer period of time.

### 2.5. Correlation of Electric Properties and Surface Wetting of LDPE/EVA Films

In order to show the correlation between the bulk electrical conductivity of the coextruded films and their wetting behavior, the electric conductivity in alternating current at a sweep frequency was measured. Due to the electrical insulation properties of these polymers, the electrical conductivity took place at high frequencies. Nonionic surfactants are more conductive than polymeric LDPE [16] and these agents had the capability to increase the conductivity of the LDPE/EVA membrane.

**Figure 5:**
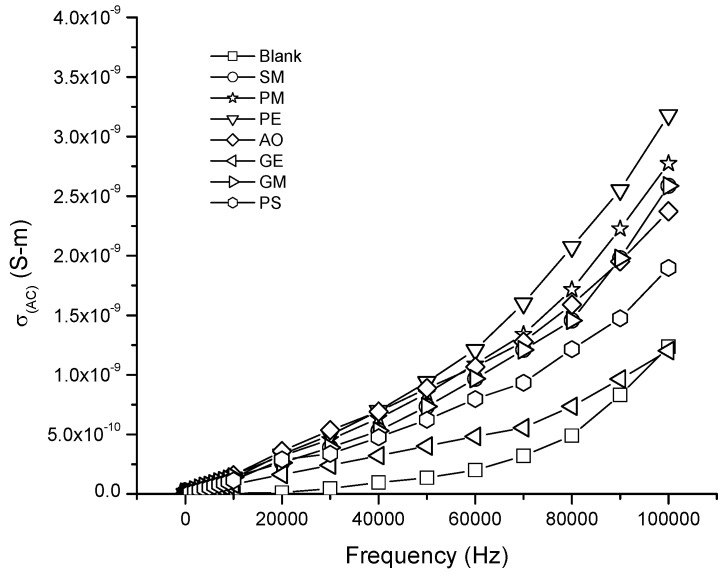
Electrical conductivity AC of LDPE/EVA coextruded film containing 3 wt % of the different nonionic surfactants.

Figure 5 shows the electrical conductivity AC of type I membranes, with 3 wt % of nonionic surfactants, in the coextruded films. For the nonionic surfactants used in the membrane, the conductivity increase occurred at high frequency. This performance was displayed at 40,000 Hz. Notice that the additives PE, PM, SM, OA, PS, GE and GM presented higher electrical conductivity (AC) than the blank.

**Figure 6:**
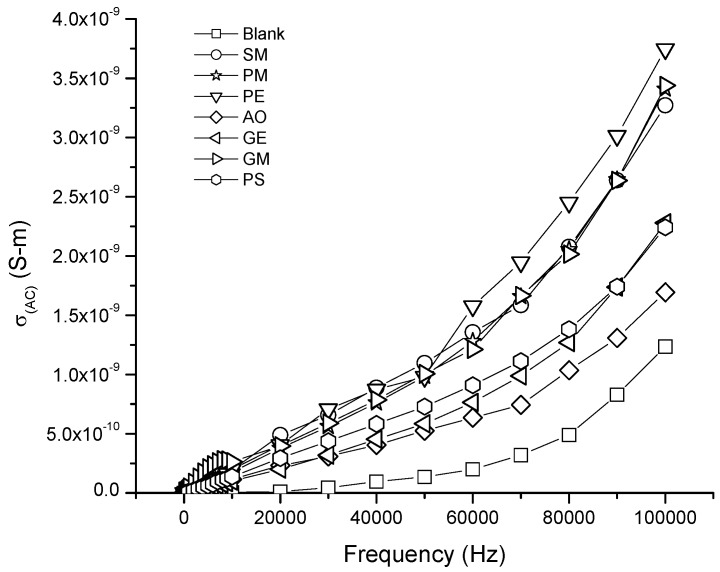
Electrical conductivity AC of LDPE/EVA coextruded film containing 5 wt % of the different nonionic surfactants.

The effect on the electrical properties of membranes when the concentration of nonionic surfactant was increased to 5 wt % (type II) membranes is shown in Figure 6. An increase in the conductivity (AC) at high concentration of the GM, PE, GE, PM, PS and SM surfactants was observed. In contrast with the type I membranes, the value of the electrical conductivity increased about 1.0 × 10^−9^ S/m. The exception was the membrane with the OA additive, since it presented a minor value of electrical conductivity (AC) at 5 wt % compared to 3 wt %.

A correlation between the electrical conductivity and the wetting permanency in the plastic membrane was found. This means that when the conductivity of the nonionic surfactant is higher, the hydrophilic affinity of the LDPE film increases. As described above, the evolution of the condensation kinetics shows a greater permanency on the anti-fog effect of type II membranes containing SM, PM, PS and GM nonionic surfactants. GE showed a short duration of the anti-fog effect and also had a low effect on the film conductivity increase.

Additionally, the OA surfactant presented a poor performance in the hot fog test and a low conductivity independently of the concentration. We can conclude that the OA compound is expelled from the film in a short time, and PE membranes do not have a correlation between high electrical conductivity (AC) and poor performance in the hot fog test, at both concentrations of 3 and 5 wt %. This exceptional case can be studied in other work.

## 3. Materials and Methods

Materials: Low-density polyethylene (LDPE) of melt flow index 8 from Dow Company (Plaquemine, LA, USA). Ethylene vinyl acetate (EVA) copolymer with 18% of vinyl acetate from Exxon Mobil Chemical (Houston, TX, USA) were used. Nonionic surfactants integrated in LDPE were kindly provided by A. Schulman de México (San Luis Potosí SLP, México) and these were: Glycerol ester (GE), Octadecanoic acid (OA), Polyglycerol ester (PE), Polyglycerol stearate; 1,2,3-Propanetriol (PS), Polyglycerol monostearate (PM), Sorbitan monostearate (SM), Glyceril monooleate (GM).

Procedure: The coextruded films were produced, with a width of 175 micrometers, in a Killion cast film extruder provided with a bi-layer die. The wetting agent was blended into the polymer during an extrusion process. The bilayer film was produced at a 70/30 (A/B) ratio. Films with 3 wt % (Type I Membrane) and 5 wt % of additive (Type II Membrane) in layer A were produced, for which one of the layers (layer A) were made with an EVA/LDPE blend such that a final amount of 15% of vinyl acetate groups is obtained. Additionally, a high concentration of wetting additive (3 or 5 wt %) is added to layer A. The other layer (layer B) is produced only with LDPE and 1 wt % of wetting additive.

Characterization: Wetting tests were realized for both A/B sides of the film consisting on Mean Contact Angle measures and a diffusion kinetic through an observation of water condensation over the film surface on a Hot fog Chamber. Additionally, the wetting performance was characterized by the correlation with the electric conductivity measurements.

Mean Contact Angle (MCA): MCA test provides information of the wetting performance due to microscopic effects related with the chemical nature of the amphiphilic compound. Contact angle measurements were recorded with a goniometer (Ramé-Hart Instrument Co., Succassuna, NJ, USA), using the sessile method [17]. This method consists on carefully placing a drop of distilled water onto the polymeric material surface using an insulin syringe and then measure the contact angle between the drop surface and the polymeric sample. Five drops (distilled water) were analyzed per film. During each measurement the software analysis takes the left and right contact angle and the average of both sides, registering values each 0.001 s until it reaches 50 measurements. On the final analysis, the data reported is the mean contact angle (MCA), i.e., the mean value of the 250 average measurements described above. Evaluation was done 30 days after the film manufacture which was considered an adequate time for the different additives diffusion to be achieved.

Fourier Transform Infrared (FTIR) Microscopy: FTIR spectroscopy was used to observe the functional groups distribution over the surface of selected films. The surface FTIR scanning was done with a Perkin Elmer Spotlight 200 FT-IR Microscopy System (Waltham, MA, USA) in the reflectance mode in order to have a superficial signal of the migration of nonionic surfactants. Spectra were collected in the range of 650–4000 cm^−1^ with 16 scans per pixel.

Condensation kinetic: The hot fog test provides qualitative information on the performance of the wettability on a plastic film surface over a specific time lapse, mostly related with the diffusion agent behavior in the membrane. This method consists on placing the film material in contact with water vapor and observe the formation or not of fog on the material. Inside the chamber, water is in contact with a thermal bath at 40 °C during the entire test. Results based on the hot fog test can be multiplied by a factor of ~4 to predict performance in the field [1]. Fog is a consequence of ordering water molecules into micelles due the poor affinity of the material with water, which can be observed as macroscopic water drops. The condensation level of the material is visually evaluated according of ratings described: (0) Zero visibility; by opaque layer of small fog droplets; (1) Very poor visibility; by opaque or transparent layer of large fog droplets; (2) Poor visibility; a complete layer of large transparent drops; (3) Good visibility; some drops randomly scattered (4) Completely transparent; a transparent film displaying no visible water [1].

Electrical conductivity of films: In non-polar films when is applied high frequency these can change to the film in conductive of alternate current (AC) [18]. In this study we will test the electrical properties in bulk of films with added different nonionic surfactants to correlate the interaction with water. The alternate current electrical conductivity was measured with an nf LCR Meter model ZM2372 (nf corporation, Yokohama, Japan). Silver paste was coated on the film surfaces to form an electrode. The LCR measurements were with 5 V in 1 Hz–100 KHz frequency range.

## 4. Conclusions

All the studied nonionic surfactants showed increased water affinity on the plastic membrane. The MCA test suggests that the wetting is poorer for a greater amount of additive. This effect could be explained by the formation of aggregates at high concentrations of additive (>5 wt %) which makes the exposition of hydrophilic functionality difficult.

From kinetic condensation by the hot fog test, we observed that using a 5 wt % concentration of nonionic surfactants increases the acceptable performance. These results suggest that membranes made of the EVA and LDPE layers prevent rapid migration or vanishing of the amphiphilic agents when the EVA layer faces the water vapor. In addition, the additives PM and SM at a 5 wt % concentration presented an acceptable performance over a long time (~3000 h).

We showed a direct correlation with the wetting performance and the electric conductivity. The best performance of wetting was obtained with the additives that increased the electric conductivity of the membrane.

An overall conclusion from our study is that a long-lasting wetting performance can be achieved with additives with a MCA value close to 30°. We have shown that films with a MCA of ~30° and a good polarization capacity (electric conductivity) have the best performance in the hot fog test. We can conclude that both tests can be used as a quick evaluation to demonstrate the anti-fog effect of the membrane.

## Figures and Tables

**Table 1:** MCA on both faces of membranes type I, formed with EVA layer (side A) and pure LDPE layer (side B) with 3% and 1% of the nonionic surfactant concentration, respectively, for each side.

ID Film	Layer A	Layer B
	MCA	MCA
blank ^1^	93 ± 2	99 ± 2
OA	6 ± 1	~0
GM	20 ± 2	27 ± 3
GE	24 ± 2	17 ± 2
SM	27 ± 6	28 ± 4
PM	33 ± 3	60 ± 5
PE	34 ± 8	64 ± 8
PS	48 ± 2	89 ± 4

^1^ Film without surfactant.

**Table 2:** Molecular weight of the studied nonionic surfactants.

ID Surfactant	Molecular Weight g/mol
OA	284.48
GM	358.56
GE	356.55
SM	430.62
PM	506.72
PE	420
PS	726.48
